# Preventing stress-related ill health among newly registered nurses by supporting engagement in proactive behaviors: development and feasibility testing of a behavior change intervention

**DOI:** 10.1186/s40814-017-0219-7

**Published:** 2018-01-08

**Authors:** Elin Frögéli, Ann Rudman, Brjánn Ljótsson, Petter Gustavsson

**Affiliations:** Department of Clinical Neuroscience, Karolinska Institutet, Nobels väg 9, 171 65 Solna, Stockholm Sweden

**Keywords:** Intervention, Nurse, Prevention, Proactivity, Recovery, Stress, Socialization, Stress-related ill health

## Abstract

**Background:**

Transitioning into a new professional role is a stressful experience with consequences for mental and physical health, job satisfaction, organizational commitment, and turnover. New registered nurses seem to be at particular risk of developing stress-related ill health during their first years in the profession. Previous research indicates that engagement in proactive behaviors may reduce this risk.

**Methods:**

With the work presented in this paper, we aimed to test the feasibility of conducting an evaluation of the effect of a behavior change intervention to prevent stress-related ill health among new registered nurses by supporting their engagement in proactive behaviors. Feasibility objectives included recruitment, randomization, data collection and analysis, participation, acceptability, and deliverability.

We tested the feasibility of evaluating the effect of the intervention as part of a transition-to-practice program for new registered nurses using a non-randomized design with one condition. The trial included a sample of 65 new registered nurses who had been working for 6 months or less.

**Results:**

The feasibility of conducting a full-scale effect evaluation was confirmed for recruitment, data collection and analysis, participation, and acceptability. It was not possible to randomize participants, but analyses of between-group differences revealed no selection bias. The time of the intervention will need to be extended to ensure the deliverability.

**Conclusion:**

With some adjustments in the study design, it is feasible to evaluate the effect of a behavior change intervention to support new registered nurses’ engagement in proactive behaviors during their transition into the new profession as part of a transition-to-practice program for new nurses.

## Background

Registered nurses are a professional group that has been recognized as experiencing high levels of stress-related ill health (i.e., burnout [1–3]). New registered nurses are at particular risk. Boamah and Laschinger [4] and Laschinger et al. [5] found that nearly half of new registered nurses in their Canadian sample experienced severe levels of symptoms of stress-related ill health. In addition, every fifth new registered nurse in a national representative sample in Sweden was found to experience extremely high levels of stress-related ill health at some point during the first 3 years in the profession [6].

Heavy workload, large emotional demands, and limited resources have been acknowledged as important sources of stress for the general nursing population [7, 8]. The same stressors have also been recognized as important for new registered nurses [9, 10]. In addition, for many new registered nurses, the experience of stress is exacerbated by the perception of oneself as inadequately prepared and unable to live up to role expectations [6, 11–14]. New registered nurses who experience symptoms of stress-related ill health are less satisfied with their job [15, 16] and have more thoughts about leaving their current place of employment or the nursing profession than other new registered nurses [6, 17, 18].

Considering the prevalence and consequences of stress-related ill health among new registered nurses in the transition from education to profession, developing methods for preventing this problem has been recognized as important [6, 16, 19]. This research should be based on a thorough understanding of how stress-related ill health is developed among new registered nurses and how it may be effectively prevented [20, 21].

### Behavioral model of development and prevention of stress-related ill health

The purpose of the stress response is to support effective management of challenging or threatening situations through physiological, emotional, and behavioral adaptations. However, if prolonged or repeated, it may have damaging effects on the physiological systems involved leading to symptoms of stress-related ill health [22, 23]. Although behavioral avoidance is essential to the adaptive management of stressors, it has been suggested that excessive engagement in avoidance behaviors contribute to the malevolent prolonged or repeated activation of the stress response [22, 24–27]. In line with this suggestion, it has been shown that stress-related ill health among new registered nurses may be modeled as a sequential-developmental process that develops progressively from initial levels of perceived stress and exhaustion as the new registered nurses engage in behaviors such as approaching work tasks in a mechanistic way and talking about work in a detached way [28].

Meta-analyses of stress management interventions have shown that behavior change programs may be effective for reducing stress-related ill health among experienced registered nurses. These interventions typically support the development of behaviors that serve the purpose of effectively managing stressful situations, as opposed to avoiding them and thereby reduce the experience of stress over time [29–31]. A similar intervention was also shown to reduce stress among nursing students [32]. However, a review of interventions for preventing stress-related ill health among new registered nurses revealed that only limited evidence is available [33].

### Proactive behaviors

Behaviors such as practicing new skills, seeking information, asking for help, monitoring and imitating the behavior of experienced colleagues, and making contact with peers for relationship building have been recognized as important for the management of challenging situations during the transition into a new profession [34–36]. Specifically, these behaviors have been positively related to processes such as mastery of occupational tasks, role clarity, and social acceptance [37, 38]. These processes have been suggested to mediate newcomers’ acquisition of the knowledge and skills required to adapt to the professional role [39]. In addition, these processes are positively related to performance, job satisfaction, and organizational commitment and negatively related to intention to leave and turnover [37, 38]. Furthermore, in a longitudinal study conducted in Sweden with a sample of 1210 new registered nurses, higher levels of the processes were found to be related to lower levels of symptoms of stress-related ill health concurrently and over time during the first 3 years in the profession (Frögéli, Rudman, Lövgren, Gustavsson; Problems with task mastery, social acceptance, and role clarity explain nurses’ burnout during the first professional years. Submitted). That is, the higher the perceived task mastery, role clarity, and social acceptance, the lower the risk of stress-related ill health. Proactive behaviors thus constitute a potential target for preventing stress-related ill health among new nurses.

### Study objectives

With the work presented in this paper, we tested the feasibility of conducting a full-scale effect evaluation of an intervention developed for preventing stress-related ill health among new registered nurses [20, 21]. The intervention was developed based on the hypothesis that stress-related ill health among new registered nurses may be prevented by increasing their engagement in proactive behaviors and that the effect of increased engagement in proactive behaviors on stress would be mediated by increased mastery of occupational tasks, role clarity, and social acceptance. The intended primary outcome of the full-scale evaluation is stress as measured by a stress and energy questionnaire [40]. The feasibility objectives of the present study included recruitment, randomization, data collection and analysis, participation, acceptability, and deliverability of the intervention.

## Methods

### Trial design

This study used a non-randomized design with one condition to investigate the feasibility of conducting a full-scale evaluation of the effects of an intervention developed to prevent stress-related ill health among new registered nurses. The trial took place at a hospital in Sweden as part of a transition-to-practice program for new nurses. Data were collected during the fall of 2015.

### Participants

All newly graduated nurses who were starting their first employment as qualified nurses and were registered to participate in the transition-to-practice program (*n* = 82) were invited to participate in the trial. Recruitment took place at an information meeting at the hospital in September 2015 where all potential participants were scheduled to attend. Information about the study was given verbally and in writing by EF and AR. A signed informed consent was required for participation in the study.

### Intervention

Prior to this feasibility trial, to develop the intervention, we collected data from semi-structured interviews with 12 nurses (10 women; 2 men) working within their first 12 months of the profession (range 2–12 months). The interviews were performed individually face-to-face by members of our research group (EF and AR plus others) with experience of conducting interviews. Each interview was approximately 60 min long. The focus of the interviews was to gather information about the challenges experienced by the new registered nurses as they entered the profession, related thoughts and emotions, and how they acted to manage the challenges. To get information-rich material, we sampled new registered nurses from different geographical regions, hospitals, and clinical specialties until saturation of information was achieved [41, 42]. The interviews were recorded using the Olympus Digital Voice Recorder WS-833 and transcribed verbatim prior to analysis by a third party with previous experience of transcribing interviews.

The analysis of the material was conducted by EF, with ongoing input from AR, BL, and PG. A deductive content analysis approach [43] was used together with the principles of functional analysis [44, 45], as well as theories from organizational socialization [46] and stress research [22, 23] to analyze new registered nurses’ engagement in proactive behaviors with the purpose of clarifying how the behaviors were learnt and maintained. We also analyzed nurses’ avoidance behaviors in situations in which proactive behaviors would have been adaptive but were not present, as previous research has shown that engagement in proactive behaviors is at times inhibited by fear of aversive events [35, 47]. Finally, as exhaustion has been shown to reduce engagement in proactive behaviors [48, 49], and some leisure activities have been suggested to protect against exhaustion [22, 23], we also analyzed the participants’ engagement in energizing leisure activities.

Central to the functional analysis are the antecedents and consequences of behaviors. Antecedents (e.g., thoughts, emotions, or situational factors) function as signals indicating that certain consequences may be expected if a behavior is engaged in. Consequences follow on the behavior and may produce changes in the rate of the behavior over time (i.e., increase or decrease the likelihood of engaging in the behavior in the future) [45, 50]. The term behavior in this context refers to overt behaviors as well as covert behaviors such as cognitive and emotional processes [44, 45].

**Fig. 1:**
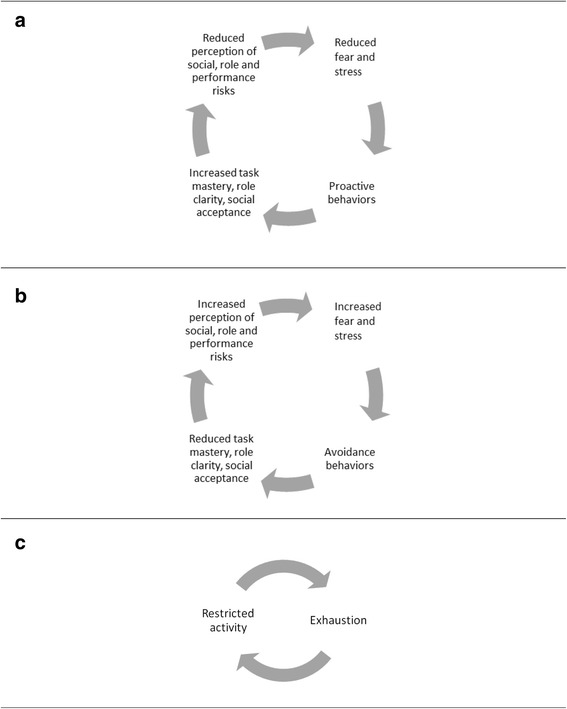
**a** Positive spiral of engagement in proactive behaviors. **b** Negative spiral of engagement in avoidance behaviors. **c** Negative spiral of restricted engagement in leisure activities

Antecedents for new nurses’ proactive behaviors (e.g., asking for help and seeking information in documents and on the web) were the experience of uncertainty, the perception of colleagues’ willingness to help, and the perception of one’s personal capability to execute the proactive behaviors. As presented in Fig. 1a, engagement in proactive behaviors led to increased task mastery, role clarity, and social acceptance, which in turn led to reduced perception of risks and stress, and facilitated future engagement in proactive behaviors. The following are statements made by the respondents about engagement in proactive behaviors.

I could ask my colleagues anything, that was really supportive. At the slightest uncertainty, I could ask a colleague instead of feeling inadequate for not knowing. (ID 1)

When I, for example, encounter a new drug, I read information about it. Or I ask a colleague if he or she has any previous experience with that drug. That way you can exchange knowledge and experience. (ID 7)

The first time [that the respondent was to do a certain task] I asked a colleague to demonstrate, so that I could see how it should be done. And then I asked if I could do it with my colleague standing by my side. And then, the next time, I did it by myself. (ID 5)

However, in situations when the new registered nurses were afraid that they would not be able to handle the responsibility that was placed on them in their new professional role, that they would make significant and dangerous mistakes, and that they would not be included in their new social group, they tended to avoid engaging in proactive behaviors (e.g., avoided asking for help when in need, avoided engaging in difficult assignments, avoided delegating tasks to assistant nurses, and avoided taking breaks). These avoidance behaviors were maintained as they were followed by the termination of fear and distress. However, although the momentary distress was reduced, engagement in avoidance behaviors led to increased fear and stress over time as skills and knowledge were not developed, relationships were not strengthened, and professional roles were blurred. This negative spiral is illustrated in Fig. 1b. The following are statements made by the respondents about avoidance of proactive behaviors.

To take on the role as a leader of the team can be a real challenge. Many of the assistant nurses have worked for many years. And then you arrive, straight out of school, and you are supposed to tell people what to do. It was stressful, and I did not dare to tell some of the assistant nurses what to do, so I ended up taking on more tasks myself than I should have done. (ID 2)

You want to be accepted by the group, you do not want to be the one who asks questions all of the time, and you do not want to be the one that your colleagues are not confident about. I mean, it is not only a matter of work, you also want to fit in socially. (ID 12)

Many times I have so much to do that I, I don’t know, I tend to realize in the morning when I am on my way back to work that, “Oh, I forgot to do this or that”. And then it feels really stressful to go back to work. You feel as if you are a lousy nurse, and as if, I don’t know, you have too much to do. And many times I believe that it is due to the fact that you have not taken a break. You are so concerned about making it all run smoothly. And then I feel that I am not very good at prioritizing my own tasks. I easily end up helping the assistant nurses, even though I should be reading up on information about the patients, or making a phone call to someone’s relatives. I try to think, “What could I do differently?”. And I believe that, if I was able to pause for a little while, that would make a difference. But it is difficult to tell someone “You have to do this because I do not have the time right now”. It feels as if I am blocked by the stress that I am experiencing. (ID 8)

I have noticed that, when it is stressful, I avoid some tasks that I would not normally avoid. And then I think, “Next time I will do it differently”. But then, the next time you are in the same stressful situation you end up doing it the same way all over again. (ID 5)

I worked longer to compensate for the fact that I could not manage my tasks in time. That took a lot of energy. It is the first thing you think about when you wake up in the morning, and the last thing you think about when you go to bed at night. I was terrified that I would make a mistake. I could not stop thinking about work. I asked myself, “Did I do everything correctly?”. (ID 12)

The new nurses furthermore declared that they had cut down on engaging in social activities, hobbies, and exercise following the start of their new profession. Exhaustion and fear of exhaustion served as antecedents for engaging in passive behaviors such as lying in bed or sitting in the sofa watching television. However, although these passive behaviors reduced the immediate experience and fear of exhaustion, the restricted engagement in leisure activities led to increased exhaustion over time.

In addition, shiftwork further contributed to the restricted engagement in leisure activities. Working day and evening shifts intermittently caused the new registered nurses to lose contact with antecedents and consequences of behaviors that they had previously engaged in, making them less likely to be engaged in further on. The negative spiral of reduced engagement in leisure activities and increased exhaustion is presented in Fig. 1c. The following are statements made by the respondents about exhaustion and engagement in leisure activities.

You are so tired all of the time. A lot of the time I go straight to the sofa when I get home from work. Then I get up, have something to eat, and then I go back to the sofa again. And then it is time to go to bed. (ID 1)

I think of my sleep more nowadays then I used to do. That I need to get my eight hours, especially when I start work early. And I think about how many hours of sleep I will get when I have worked late and will start early the next day. And if I am going out I think “If I go out, when do I have to be back home to get the number of hours of sleep that I need”, or something like that. Instead of just thinking “Sure, let’s go to the movies”. That is rather sad. (ID 5)

Most people want to meet up during the weekend and then you have to work. So you prefer to meet up during the week, but that is also tricky because other people need to get up early to go to work, while you may work the evening shift and have a late morning. The weekends are the most affected. The fact that you can’t participate when people meet up. That is really, really sad. (ID 1)

It’s a challenge to engage in leisure activities. For example, enrolling in a class that is planned for a specific night every week is not possible because you do not know if you will be scheduled for work on that night or not. It is a challenge to do something on a set time every week since one’s schedule is always changing. (ID 5)

The development of the intervention was based on these analyses suggesting that, to facilitate new registered nurses’ proactivity, it is important that their engagement in avoidance behaviors is reduced and that engagement in leisure activities is increased. Behavior change models show that avoidance behaviors may be changed by intentionally approaching feared situations. If an individual approaches a previously avoided situation and notices that the feared consequences are not realized, the learnt avoidance behaviors begin to extinguish as memories of benevolent experiences in previously feared situations are acquired. This behavior change technique is known as systematic exposure [26, 51]. The challenge in getting this positive spiral in motion is refraining from engaging in avoidance behaviors when in contact with antecedents signaling that those behaviors would be followed by the reduction of fear. An additional behavior change technique referred to as action planning is used to overcome this challenge [52].

Behavioral activation is a behavior change model that was developed for use in the treatment of clinical depression [53]. According to this model, depression develops when people’s actions are more likely to be met by aversive than appetitive consequences and people thus come to restrict their behavioral repertoire. As a consequence, engagement in behaviors that are followed by appetitive consequences is also restricted. By using a behavior change technique referred to as reinforcing approach behaviors together with action planning, people may come to re-engage in behaviors that are followed by appetitive consequences [53]. The analysis suggested that these behavior change techniques may similarly be useful for supporting new registered nurses’ engagement in leisure activities.

Involvement of key stakeholders has been shown to improve effects of interventions [20, 54]. Therefore, after the analysis but prior to the feasibility trial, the manual of the intervention was read through by a nurse who had worked for almost 3 years. It was then used in individual contacts with three nurses during their first 3 months of working professionally (in 1-h meetings every other week). Finally, the core concepts of the intervention were used in four separate workshops of 2 h with a total of 65 new registered nurses. The workshops were conducted in a county in Sweden separate from the one included in the feasibility trial.

At each step of this validation process, we investigated the nurses’ recognition of the antecedents and consequences of proactive and avoidance behaviors, as well as their recognition of the restriction in leisure activities and whether or not they found the proposed techniques for behavior change interesting and feasible. Following these tests, we refined and finalized the manual for the feasibility trial to further increase its acceptance.

For the feasibility trial, the intervention was included in the schedule of a transition-to-practice program for new registered nurses. It consisted of two sessions of 2 h (i.e., in total 4 h per group) and took place at the hospital. The new registered nurses participated in the sessions in groups of approximately ten individuals. EF who is a licensed psychologist led all the sessions using the structured manual. The participants were given pamphlets covering the content of the sessions as well as worksheets for pen-and-paper exercises and homework assignments. No powerpoint presentations or other media were included in the intervention, but a whiteboard was used in both sessions. The full manual of the intervention can be obtained by contacting EF.

The work in session 1 was focused on increasing leisure activities using the behavior change technique action planning and reinforcing approach behaviors. The session started with an overview of the intervention program and a discussion about the stress response, work-related stress, and stress-related ill health. The participating nurses were invited to share their thoughts on the subjects.

Next, increasing leisure activities was presented as one way to reduce the risk of stress-related ill health, and the participating nurses worked individually with a pen-and-paper exercise. Finally, the work in the session was summarized and the new nurses were supported in formulating behavior change goals on the subject of increasing engagement in leisure activities. These behavior change goals constituted the homework assignment from session 1 to session 2.

The work in session 2 was focused on reducing engagement in avoidance behaviors and increasing engagement in proactive behaviors using action planning and systematic exposure. First of all, the work from session 1 was repeated and experiences from the homework assignment were discussed. In addition, the participating nurses were encouraged to formulate new behavior change goals on the subject of increasing engagement in leisure activities building on their experience from the previous week.

Next, avoidance behaviors were discussed in relation to stressful situations, including their short- and long-term consequences. The new nurses were supported in conducting individual analyses of engagement in avoidance behaviors in a pen-and-paper exercise. In addition, they were supported in identifying suitable proactive behaviors to engage in as alternatives to the avoidance behaviors. The challenges of engaging in proactive behaviors in situations where one has previously engaged in avoidance behaviors were discussed, together with the principles of systematic exposure. Finally, the new nurses were encouraged to formulate new behavior change goals with the purpose of reducing engagement in avoidance behaviors and increasing engagement in proactive behaviors. Before the last session was over, the content of the two sessions was revised.

### Feasibility criteria

There is not yet a consensus on what should be the focus of study in feasibility trials and what should be the feasibility criteria [55]. In this study, we investigated the feasibility of recruitment, randomization, data collection and analysis, participation, acceptability, and deliverability of the intervention and we decided that the effect evaluation would be considered feasible to conduct if:

1. Seventy percent (or more) consented to participating in the feasibility study
2. It would be possible to randomize participants to experimental and control groups using simple randomization with no systematic differences between the groups at baseline
3. All of the self-report measures would be successfully distributed and 70% of the participants would respond at post-intervention
4. The self-report measures would be reliable (Cronbach’s alpha > 0.70 or mean inter-item correlation > 0.20 at baseline) and sensitive to change from pre- to post-intervention and follow-up (proportion of variance over time that is not attributed to stable differences among individuals for the process and behavior variables, i.e., an intra-class correlation of about 0.50)
5. Seventy percent of participants would participate in both sessions of the intervention
6. Seventy percent of the participants would rate their acceptance of the intervention as a four or five on a 5-point Likert scale
7. The intervention could be delivered according to protocol during the allocated time.

### Data collection and analysis

We used a number of different methods to evaluate the feasibility objectives. According to Craig et al. [20] and Tebes et al. [21], randomized designs should always be considered when evaluating the effect of interventions because it is the most effective design to prevent the risk of selection bias leading to systematic differences in the samples being compared. However, it has been recognized that randomization is not always feasible. In such cases, evaluating the likelihood of selection bias is important to avoid incorrect interpretations of results. Thus, although this study did not aim to evaluate between-groups effects, we included a random allocation of participants to two groups in the design to investigate the feasibility of this procedure as well as the likelihood of selection bias when using this procedure. Allocation to groups was performed by the hospital as new nurses were registered to the transition-to-practice program (sequential allocation to the two groups, ratio 1:1). We evaluated the likelihood of selection bias using independent sample *t* tests of the differences between the groups on all study measures.

We distributed the self-report measures using electronic surveys [56] at baseline (time 0), just prior to the intervention (time 1), 1 week after the completion of the intervention (time 2), and at 5 weeks after the intervention (time 3). With the exception of the baseline survey, all surveys were distributed electronically using e-mail addresses that the participants reported when entering the study (the participants responded to the baseline survey electronically using a URL that was presented at the recruitment session). The surveys were closed, that is, each respondent received a survey with a specific identification number, and could only be responded to one single time. One reminder per survey was given to participants who had not responded after 1 week. Data on distribution and responses was extracted from the electronic survey tool.

We evaluated the reliability of the self-report measures (perceived stress [40] [primary outcome], mastery of occupational tasks [57], role clarity [58], social acceptance [57] [process measures], engagement in proactive behaviors [developed based on [59]], and avoidance of proactive behaviors [developed for this study] [behavior measures]) using Cronbach’s alpha and analysis of mean inter-item correlation with data from time 0. A Cronbach’s alpha above 0.70 is indicative of a good reliability in the measure. However, the estimate is sensitive to the number of items included in the analysis. The mean inter-item correlation analysis complements the analysis by providing an estimate for the mean correlation between the items included in the measure. A mean inter-item correlation of 0.20 suggests that there is sufficient homogeneity in the items [60]. With the exception of the two behavioral measures that were developed for this trial, all measures were previously validated in Swedish working populations. All measures were coded so that a higher score indicated a higher value of the construct being measured (e.g., a higher level of stress or a higher level of role clarity).

The variability of the process and behavior variables were evaluated using the intra-class correlation computed based on the variance components of a multilevel unconditional means model including data from time 1, time 2, and time 3. The intra-class correlation coefficient is a numerical representation of the proportion of the variation in a variable that is attributable to differences between individuals as compared to differences within individuals [61]. A measure for which a large proportion of the total variance lies between subjects (i.e., the intra-class correlation is close to 1.0) has limited applicability as a measure to evaluate change in relation to an intervention.

The evaluation of the percentage of subjects who participated in both sessions of the intervention was based on data on participation that was recorded at each session. We evaluated the participants’ acceptability of the intervention using three questions that were administered electronically immediately after each session and answered on a 5-point Likert scale. We computed the percentage of participants scoring 4 or 5 on the scale and analyzed this separately for each session and item.

Finally, we analyzed the feasibility of delivering the intervention based on comments about the deliverability of the material that were recorded by EF at each session. Throughout all the analyses in this study, we used the intent-to-treat approach including all study participants.

### Ethical aspects

The study was approved by the ethical committee at the Karolinska Institutet (2014/1531-31/5) and was conducted in accordance with the Helsinki Declaration of ethical principles for medical research involving human subjects [62]. Participation in the trial was voluntary, and a signed informed consent form was required. The participants were informed that their employer would not gain access to any information that could be connected to individual participants. All registered nurses participating in the transition-to-practice program were scheduled to participate in the intervention (i.e., regardless of them choosing to participate in the feasibility trial or not). Subjects who did not wish to participate in the intervention would have been granted permission not to do so; however, no one requested this. No incentives were given for participation.

## Results

### Participant flow

**Fig. 2:**
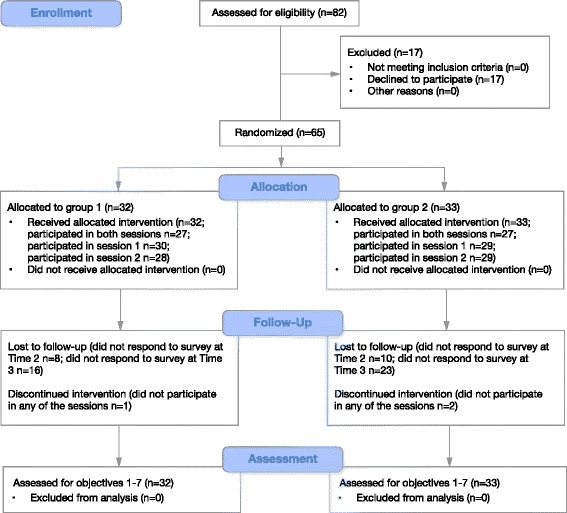
Flow of participants

The flow of the participation is presented in Fig. 2 using an adapted version of the CONSORT diagram [63, 64]. Sixty-five new registered nurses consented to participate (79.3%). The mean age of the study sample at baseline was 25.95 years (SD = 4.02, range 22–45), and 86.2% were females. One in ten (10.8%) had held a position at their current workplace prior to the nursing education, and 16.9% had held a position at their current workplace during the nursing education. At the time of their participation in the intervention, 95% of the sample had worked as nurses for 6 months or less (one nurse had worked for 7 months, two nurses had worked for 10 months, and two nurses did not report the exact date that they started their employment).

### Baseline data

In Table 1, all self-report measures are presented together with sample means at time 0. In addition, the correlations of the scales at time 0 are presented in Table 2.

**Table 1:** Names, references, number of items, scale ranges, and mean and standard deviations of measures at baseline (time 0)

	Variable	Reference	Items	Scale	*M* (SD)
	Primary outcomes				
1	Stress	Kjellberg and Iwanowski 1989	6	1–6	3.75 (0.87)
	Process variables				
4	Role clarity	Dallner et al. 2000	3	1–5	3.88 (0.60)
5	Social acceptance	Aurell et al. 2015	2	1–5	4.09 (0.82)
6	Mastery of tasks	Aurell et al. 2015	2	1–5	3.60 (0.74)
	Behavior variables				
7	Proactivity	Reeves, 2013	4	1–5	3.15 (0.67)
8	Avoidance of proactivity	Developed for this study	2	1–5	1.96 (0.80)

*M* mean, *SD* standard deviation, *scale range Stress* 1 = not at all to *6* = very much, *scale range Role clarity, Social acceptance, Mastery of tasks/Proactivity, and Avoidance of proactivity* 1 = never to 5 always (measured at time 1 [not included at time 0])

**Table 2:** Correlations at baseline (time 0)

	Variable	1	2	3	4	5	6
	Primary outcomes						
1	Stress	1					
	Process variables						
2	Role clarity	− 0.16	1				
3	Social acceptance	− 0.50*	0.14	1			
4	Mastery of tasks	− 0.32*	0.21	0.26*	1		
	Behavior variables						
5	Proactivity	− 0.02	0.22	0.10	− 0.15	1	
6	Avoidance of proactivity^a^	0.24	− 0.19	− 0.25	− 0.15	0.05	1

*p < 0.05

^a^Measured at time 1 (not included at time 0)

### Outcomes

The results of the evaluation of the feasibility objectives are presented below. Each result is commented with reference to the feasibility criteria.

### Recruitment

A total of 82 new registered nurses were registered in the transition-to-practice program in which this trial was incorporated. Sixty-five of these gave their informed consent to participate in the feasibility trial and provided baseline data (79.3%). Thus, the feasibility criterion of at least 70% was met.

### Randomization

It was not possible to randomize participants using simple randomization. The reason for this was that more than one new registered nurse from some clinical wards participated in the transition-to-practice program. To avoid staffing problems on these clinical wards, these registered nurses were allocated to separate groups in the program. The managers of the clinical wards were given the possibility of choosing which group their new employees would be assigned to (group 1 or group 2). Of the 65 new registered nurses who consented to participate in the feasibility study, 32 belonged to group 1 and 33 to group 2.

There were no significant differences in the study variables at time 0 among the participants of the two groups (stress: *t*(62) = − 0.96, *p* = 0.340, 95% CI of difference between groups = − 0.64 to 0.22; role clarity: *t*(62) = 0.27, *p* = 0.785, 95% CI = − 0.26 to 0.35; social acceptance: *t*(62) = 0.92, *p* = 0.362, 95% CI = − 0.22 to 0.60; mastery of tasks: *t*(62) = − 0.76, *p* = 0.449, 95% CI = − 0.51 to 0.23; proactivity: *t*(62) = 0.89, *p* = 0.379, 95% CI = − 0.19 to 0.48; Avoidance of proactivity: *t*(53) = 0.03, *p* = 0.976, 95% CI = − 0.43–0.44). Thus, although a constraint in the making of the groups was needed and hence the feasibility criterion of simple randomization was not met, this study showed that it was possible to allocate the new registered nurses to two groups of equal size without introducing a selection bias.

### Data collection

Collecting data using the electronic surveys was practicable and all of the surveys were sent out as planned. The response rate was 72.3% at time 2 which was above the set level indicating that, in terms of data collection, an effect evaluation study would be feasible.

### Appropriateness of measures

The reliability estimates (*α* and mean inter-item correlation) of the study variables are presented in Table 3 together with the intra-class correlation coefficients. With the exception of the measure of proactivity, the reliability of the scales was satisfactory. It is possible that the proactivity items would be better used as single items.

**Table 3:** Reliability (*α* and mean inter-item correlation) and variability (intra-class correlation) of study variables

Variable	*α*	MIIC	ICC
Primary outcomes			
Stress	0.87	0.53	–
Process variables			
Role clarity	0.77	0.53	0.63
Social acceptance	0.70	0.56	0.45
Mastery of tasks	0.68	0.52	0.63
Behavior variables			
Proactivity	0.50	0.21	0.59
Avoidance of proactivity	0.73	0.58	0.46

*α* coefficient alpha, *MIIC* mean inter-item correlation, *ICC* intra-class correlation

The proportion of the total variance in the variables that were associated with differences between participants rather than within participant ranged from 0.45 to 0.63 as indicated by the intra-class correlation coefficients presented in column 4. Thus, the process and behavior variables were found to be sensitive to within-individual variation and should be able to show change following the intervention in the effect evaluation study.

### Participation in sessions

Fifty-four of the new registered nurses participated in both sessions (83.1% of the sample) meeting the criterion for feasibility (Fig. 2). Eight participants missed one of the two sessions, and three participants did not partake in any of the sessions. The main reasons for non-attendance was being sick and being required to work on the clinical ward although scheduled for the transition-to-practice program.

### Acceptability of intervention

The results concerning the acceptability of the intervention are presented in Table 4. Ratings are given for sessions 1 and 2 separately. The 5-point scale used to indicate level of agreement with the statements ranged from “Do not agree at all” to “Completely agree.” The number of participants answering each question is presented in the third column of the table. As indicated in the last column, the percentage of the respondents who scored 4 or 5 on the questions ranged between 76 and 98% for the two sessions. These results indicate that the acceptance of the intervention among the participating new registered nurses was above the feasibility criteria. Almost all participants found the work in sessions 1 and 2 to be relevant for them both as a nurse and as an individual.

**Table 4:** Percentage of participants who scored 4 or 5 on questions about acceptability of the intervention (*n* = number of participants answering each question)

Question	Session	*n*	≥ 4 (%)
I believe the work in this session is relevant for professional nurses	1	52	98
	2	49	84
I believe the work in this session is relevant for me as an individual	1	52	96
	2	49	88
Overall I am satisfied with this session	1	49	94
	2	49	76

### Deliverability

The allocated time of 2 × 2 h was too short to cover the material without rushing, and therefore, the behavior change techniques were not implemented in the most optimal way. More time should be given to the presentation of the work of behavioral avoidance as well as the planning of behavior change in relation to leisure activities and avoidance behaviors. In addition, a third session in which it is possible to continue the work from session 2 should be added to the design as this work could not be followed up within the current structure.

## Discussion

The results of this study showed that with regard to recruitment, data collection, analysis, participation, and acceptability, a full-scale evaluation of the effect of the intervention as part of a transition-to-practice program for new registered nurses would be feasible. However, the results furthermore showed that it will not be feasible to randomize participants to experiment and control groups using simple randomization. Participants’ clinical placements will have to be taken into consideration to avoid introducing staffing problems on their wards. It is well recognized that it is not always possible to randomize participants using simple randomization [20]. According to the results of this feasibility trial, this constraint should not introduce a selection bias and hence should not increase the risk of incorrect interpretations of results.

In addition, it was not possible to deliver the intervention in accordance with the manual during the allocated time of 2 × 2 h. Limitations in the deliverability of interventions is a commonly recognized problem of effect evaluations [20]. To amend this problem, we suggest that the time allocated for the intervention should be extended for the full-scale effect evaluation.

With these amendments in the design, the results of this feasibility trial suggest that it would be possible to move forward to the next stage of the process of development to investigate the effect of the intervention in a full-scale trial. However, although the data collection feasibility criteria of 70% response rates at time 2 was fulfilled, the response rate was only just above the limit. In addition, the psychometric properties of the self-report measure of proactivity were not completely satisfactory. Therefore, to ensure that the effect of the intervention may be properly evaluated, a qualitative investigation using interviews with participants should be added to the design. Using qualitative methods in addition to quantitative methods in the design of effect evaluations has been suggested as a potentially necessary strategy to evaluate variables such as barriers to participation [20].

In a review of interventions for preventing occupational stress in healthcare workers, it was concluded that studies need to include at least 60 participants in each group to be able to evaluate between-groups effects [31]. Considering the response rate in the present study, at least 166 participants (i.e., 83 per group) should be included in the effect evaluation to ensure the availability of data from 60 participants per group at time 1 and time 2. Given the proportion of new registered nurses invited to the present study who consented to participate (79.3%), at least 208 new registered nurses should be invited to participate in the effect evaluation.

Based on the analysis of new nurses’ engagement in proactive behaviors, the intervention was designed to focus on reducing avoidance behaviors and increasing engagement in leisure-activities. This is in line with previous research showing that maladaptive engagement in behaviors that serve the function of avoiding feared events contribute to the development of stress-related ill health [22, 24–28] and that proactive behaviors facilitate new professionals’ role adaptation and their management of challenges [34–36]. Duchscher [65], in line with the present study, suggested that new registered nurses engage in behaviors to avoid being exposed as incompetent, making mistakes, not living up to expectations, and being rejected by their new colleagues. The negative spiral of engagement in avoidance behaviors has similarly been identified in a recent review of newcomer socialization and stress [36].

In addition, it has previously been shown that newcomers’ engagement in proactive behaviors is inhibited by the experience of exhaustion [48, 49]. This has typically been linked to organizational factors such as high demands and time pressure making individuals too tired or too stressed to engage in proactive behaviors [48]. However, in the process of developing the intervention, we identified a restriction in leisure behaviors engaged in by the new registered nurses that importantly contributed to the new registered nurses’ exhaustion. Changes in leisure activities related to exhaustion was similarly found in Duchscher [65]. We found that exhaustion functioned as an antecedent for engaging in passive behaviors such as excessively watching television and sleeping. The problem was further exasperated by loss of antecedents and consequences of engaging in energizing leisure activities. The results are in line with theories of stress suggesting that engagement in active forms of recovery may protect against exhaustion [22, 23].

The value of person-directed interventions to reduce the risk of stress-related ill health among nurses has similarly been recognized elsewhere [29–31]. The high level of acceptability of the intervention in the present paper supports this idea. However, the focus on the behaviors of the new registered nurses in this study is not to be interpreted as implying that a person-directed intervention, although potentially valuable, would be a sufficient solution to the ubiquitous problem of stress-related ill health among new registered nurses. Interventions addressing the organization are clearly also important and may be effective (e.g., [66]).

Indeed, in the development of the intervention, it was recognized that perceived support from colleagues was one antecedent for engagement in proactive behaviors. This result is in line with previous research identifying co-worker and management support as an important factor affecting the establishment of newcomers [13, 14, 34, 35, 37, 65, 67, 68]. It has been suggested that an important question for future research concerns the interaction of factors supporting new professionals’ socialization at the individual level and the organizational level [34]. In line with this suggestion, it would be interesting to investigate the effect of this present intervention in combination with a behavior change model that would target the behaviors of the experienced nurses. From a functional analytic point of view, if the experienced nurses were to encourage the new registered nurses to engage in proactive behaviors as well as acknowledge their efforts in doing so, this would be expected to increase the likelihood of engagement in proactive behaviors by the addition of both antecedents and consequences.

## Conclusion

In sum, this study suggests that it would be feasible to conduct a full-scale evaluation of the effect of the intervention developed for preventing stress-related ill health among new registered nurses. Conducting a full-scale effect evaluation as part of an ongoing program for new professionals clearly presents some important challenges. By first investigating the feasibility of the study design, potential problems may be amended, resources may be conserved, the validity of the results is strengthened, and the risk of conducting a type II error is reduced. Devoting time to the development and feasibility testing of an intervention as opposed to primarily focusing on the effect evaluation is expected to result in stronger interventions that are more easy to evaluate, more likely to be implemented, and more worthwhile to implement [20].

### Limitations

In the development of the intervention, we decided that 12 new registered nurses was a sufficient sample for the analysis when no new information was provided in the interviews. It is possible that we would have gained additional information by including additional participants. However, the validity of the results and model were confirmed by a total of 69 new registered nurses and the new registered nurses in the feasibility trial found the intervention to be both acceptable and valuable. Therefore, it seems unlikely that additional participants in the interview phase would have resulted in a significantly different analysis and model.

The purpose of a feasibility study is to answer the question of whether a full-scale evaluation can be done or not. Some issues of relevance to this question were not addressed in this study. We did not investigate training needs and competence required to lead the intervention. All sessions were led by the same licensed psychologist. This trial was also limited as we did only include one hospital (it is possible that other problem would have been present in other organizations), data on adherence to the manual was only self-reported (future trials should include video or audio recordings of the sessions), and we (EF, AR, BJ, PG) developed and gave the intervention, as well as evaluated the trial. In addition, we did not investigate potential harms of the intervention.

### Generalizability

Conducting the feasibility trial as part of an ongoing transition-to-practice program ensured the ecological validity of the results. The full-scale effect evaluation of the intervention for preventing stress-related ill health among new registered nurses has been initiated [69]. The results of that trial will demonstrate if the intervention is effective in preventing stress-related ill health among new registered nurses and if the effect is mediated by increased mastery of task, role clarity, and social acceptance. If the intervention is found to be effective and it is successfully implemented, the work presented in this paper may contribute importantly to reducing the prevalence and consequences of stress-related ill health among new registered nurses in the transition from education to profession. Considering the general nature of avoidance of proactive behaviors when transitioning into a new profession [35], it seems likely that the principles of this intervention developed for new nurses could also be useful for other professional groups (e.g., teachers, social workers, doctors).
